# Health of the South

**Published:** 1843-11

**Authors:** 


HEALTH OF THE SOUTH.

While fevers of a highly malignant grade were prevailing in many parts of the South-West, during the past warm season, other districts were almost wholly exempt from febrile complaints of every character. In a pretty extensive scope of practice, in Rutherford county, in Tennessee, we did not meet with a single case of bilious remittent fever, from March till the middle of October, and a medical friend, at Murfreesborough, assured us that he had not treated a single case of the disease during the season. Intermittent fever appeared in some neighborhoods, in scattering cases, but was not prevalent in any part of the county. So healthy a season was, perhaps, never known in that region of the State. The character of the season is summed up in the following condensed statement, communicated to the Nashville Whig, by Professor Hamilton, of the Nashville University:

"The late month of September has far surpassed the corresponding months of the years immediately preceding in respect both to heat and moisture. Its mean temperature was 75.445 with a range of 40 degrees, from 53 to 93. It was warmer than September 1834 by 7¾ degrees, 1835 by 14—1836 by 2¾—1839 by 9⅞—1840 by 9—1841 by 4¾—1842 by 3⅗, and exceeded their average by 6.48. It even surpassed the late months of June and August, by about ½ a degree, and was only 4 degrees below July. As compared with the summer of 1835, it had a higher mean temperature by about 2 degrees, was about equal to that of 1842, and only one degree lower than the average summer temperature from 1834 to the present time, if we except the years 1837 and 1838. Of these two years I am unable to speak for want of a record.

"The amount of rain during the month was 8.61 inches—of this 3.22 inches fell on the 13th day in 14 hours, and 3.04 inches on the 29th and 30th in 27 hours. The whole amount during the 9 months of the present year is 43.35 inches, of which January gave 3.79—February 2.93—March 4.29—April 4.65—May 4.04—June 4.87—July 4.23—August 5.94, and September 8.61. The monthly average is about 5 inches.

"The mean degree of humidity of the air for the month was 79 inches, a scale embracing 100 degrees, and the minimum was 12, as observed on the evening of the 30th. In this scale the greatest degree of moisture is 0 and the highest degrees 100. September 30th, was the dampest day that has occurred since the 10th of February last, when the Hygrometer stood at two."

We hope our correspondents who have practised among the fevers of this season will favor us with an early account of them, as of the weather prevailing at the time. Y.

